# Methods used in early value assessments for nice: a scoping review

**DOI:** 10.1017/S0266462325100433

**Published:** 2025-09-08

**Authors:** Eugenie Evelynne Johnson, Giovany Orozco-Leal, Madeleine Still, Nicole O’Connor, Lakshmi Jayachandran, Tomos Robinson, Nick Meader, Ryan P.W. Kenny, Sheila A. Wallace, Sonia Garcia Gonzalez-Moral, Luke Vale, Rosalyn Parker, Stephen Rice, Gurdeep S. Sagoo, Fiona Pearson

**Affiliations:** 1 Evidence Synthesis Group, Population Health Sciences Institute, Faculty of Medical Sciences, Newcastle University, Newcastle upon Tyne, UK; 2 NIHR Innovation Observatory, Population Health Sciences Institute, Faculty of Medical Sciences, Newcastle University, Newcastle upon Tyne, UK; 3 Health Economics Group, Population Health Sciences Institute, Faculty of Medical Sciences, Newcastle University, Newcastle upon Tyne, UK; 4 Health Economics, London School of Hygiene and Tropical Medicine, London; UK; 5 External Assessment Group, Northern Medical Physics and Clinical Engineering, Newcastle upon Tyne Hospitals NHS Foundation Trust, Newcastle upon Tyne, UK

**Keywords:** methods, systematic review, models, economic, economic evaluation

## Abstract

**Objectives:**

The National Institute for Health and Care Excellence (NICE) in England introduced early value assessments (EVAs) as an evidence-based method of accelerating access to promising health technologies that could address unmet needs and contribute to the National Health Service’s Long Term Plan. However, there are currently no published works considering differences and commonalities in methods used between Assessment Reports for EVAs.

**Methods:**

This rapid scoping review included all completed EVAs published on the NICE website up to 23 July 2024. One reviewer screened potentially relevant records for eligibility, checked by a second reviewer. Pairs of independent reviewers extracted information on the methods used in included EVAs using a prepiloted form; these were checked for accuracy. Data were described in graphical or tabular format with an accompanying narrative summary.

**Results:**

In total, seventeen EVA Reports of sixteen EVAs were included in this scoping review. Five Reports did not specify how many reviewers undertook screening, whereas five did not report data extraction methods. Five EVAs planned to conduct meta-analyses, nine planned narrative syntheses, and seven planned narrative summaries. Eleven conceptual decision models were presented, with available evidence used to construct cost-utility analyses (*N* = 5); cost-effectiveness analyses (CEAs; *N* = 4); a mix of CEAs and cost-consequence analyses (CCA; *N* = 2); one CCA; and one cost-comparison.

**Conclusion:**

Future EVA Reports should enhance the transparency of the methods used. Furthermore, EVAs could provide opportunities for the adoption of innovative methodological approaches and more flexible communication between EVA authors and key stakeholders, including patients and clinicians, companies, and NICE.

## Introduction

In England, the National Institute for Health and Care Excellence (NICE) produces health technology appraisal guidance that makes recommendations on the use of health technologies within the National Health Service (NHS) (1). Technology appraisal guidance from NICE can cover: medicines; medical devices; diagnostic techniques; surgical procedures; and health promotion activities (1). To achieve timely patient access to health technologies, NICE have piloted multiple initiatives both across and within health technology program.

Early value assessments (EVAs) were introduced as an evidence-based way of accelerating access to promising diagnostics and medical technologies that could potentially address unmet needs and contribute to the NHS Long Term Plan (2). The interim statement on EVAs outlines the key intentions of EVAs: to identify the available evidence on the technologies; explore if technologies could address the identified unmet need; to identify important evidence gaps to direct evidence generation; and to assess if technologies should be used while further evidence is being generated (3). During the EVA process, Evidence and External Assessment Groups (EAGs), which are independent from NICE and health technology developers (4), are asked to produce an Assessment Report on technologies identified through NICE’s topic selection and scoping processes. This Assessment Report is released to stakeholders for discussion prior to committee meetings and feeds into the decision-making process. Overall, the process of producing the Assessment Report takes between 8 and 10 weeks (3). Health technologies evaluated using an EVA can either be conditionally recommended for early use in the NHS while further evidence is generated, recommended in research or not recommended for use (3). Evidence generation plans can also be developed to support technology developers in producing the evidence needed for NICE to be able to provide a clear recommendation about a technology’s future routine use within the NHS (5).

An *Early value assessment interim statement* [PMG39] from the “test and learn” phase was published in December 2022 (3). The interim statement outlines the high-level process and methods for EVAs and how they are adapted from *NICE health technology evaluations: the manual* [PMG36] (6). The statement is not intended to be prescriptive, nor does it give an overview of the methods used by EAGs to develop reports for EVAs. In November 2023, NICE’s Decision Support Unit (DSU) published a report providing guidance to EAGs needing to analyze economic evidence within the nine-week time frame of an EVA alongside the other resource limitations under which EVAs are conducted (7). The DSU report recommended conducting a pragmatic review of existing cost-effectiveness models to help the EAG obtain an existing coded model, or propose a model of the decision problem to be populated by ongoing or future data collection (i.e., a conceptual model), with ideally an accompanying simplified coded model (7).

Topics initially identified as priority areas for health technologies to be addressed by EVAs were: mental health; cardiovascular disease (specifically, predicting the risk of heart failure); early cancer detection; and technologies to increase healthcare capacity (5). Seven technologies recommended for early adoption into the NHS through the EVA program have since received £7.8 million from the National Institute for Health and Care Research and Office for Life Sciences to gather real-world evidence to accelerate a recommendation for unconditional adoption within the NHS (8). EVAs will continue following the pilot and, as such, the need to understand approaches to rapidly assessing evidence, identifying gaps and planning evidence and generation, particularly for technologies with immature evidence bases, remains.

NICE have been consulting on a new HealthTech program manual, which will include updated guidance on conducting clinical evidence reviews and economic evaluations, and modelling for EVAs (9). To further enhance transparency and robustness in future EVAs, we also need to understand more about the methods that have been used in EVAs to date. However, to our knowledge, no published information considers the approach to, and differences and commonalities, in the methods used to conduct EVAs between reports. Henceforth, this rapid scoping review aims to identify and describe evidence synthesis and health economic modelling methods used within EVAs.

## Methods

This rapid scoping review was conducted in accordance with the JBI methodology for scoping reviews (10). A full protocol for the review was prospectively published on the OSF on 22 July 2024 (11). Differences between protocol and review are detailed in Supplementary Material 1, whereas a full Preferred Reporting Items for Systematic Reviews and Meta-analyses extension for scoping review (PRISMA-ScR) checklist is presented in Supplementary Material 2 (12). Throughout this paper, “report” refers to Assessment Reports written by EAGs to inform the NICE decision-making process within EVAs.

### Eligibility criteria

We considered all completed reports for EVAs carried out by EAGs to support the potential adoption of health technologies in the NHS and published on the NICE website, regardless of the target population, intervention, or outcomes, determining the scope of the EVA. We excluded all other forms of NICE guidance (e.g., guidelines, single technology appraisals, multiple technology appraisals, medical technologies guidance, diagnostic guidance, interventional procedure guidance, highly specialized technologies guidance, late-stage assessment). Ongoing EVAs at the time of identification of evidence were noted but not formally included in the synthesis.

### Search methods

Reports of published and ongoing EVAs were identified by searching guidance on the NICE website using the terms “early value assessment,” “health technology evaluation” and “health technology assessment” on 23 July 2024, with no date or language restrictions. Results of the searches were downloaded into a Microsoft Excel spreadsheet for screening. Full details of the searches are described in Supplementary Material 3.

### Source of evidence selection

One reviewer (EEJ) assessed the results of the search. A second reviewer (GO-L) checked the extracted records for relevancy; had they arisen, any disagreements would have been discussed and resolved with a third reviewer if needed. The flow of literature is presented in PRISMA-ScR flow diagram (12).

### Data extraction

A data extraction form was developed within Microsoft Excel. The data items for the initial extraction are presented in Supplementary Material 4. Broadly, these items included: general report characteristics; intervention; population; specific methods used to identify clinical effectiveness evidence; methods used to identify economic evidence, including whether economic modelling was used; and whether patient and public involvement and engagement (PPIE) was specifically undertaken by the EAG.

To ensure accuracy of extraction and to suggest any minor changes where appropriate, the data extraction form was initially piloted on 10 percent of included reports by pairs of independent reviewers (EEJ and MS for clinical effectiveness; GO-L and TR for economic evidence). Modifications to the data extraction form following piloting are detailed in Supplementary Material 1.

Following piloting, the remaining data were extracted from reports included in the scoping review by one reviewer (either EEJ and MS for clinical effectiveness, and GO-L for economic evidence). A second reviewer (either EEJ, NOC, MS, or LJ as appropriate) checked these for accuracy. Where published EVA reports had been conducted by members of the review team, data extraction and checks were undertaken by independent reviewers to avoid any potential bias. Any disagreements that arose between the reviewers were resolved through discussion.

All relevant documentation (e.g., report, correspondence and addenda) were read and extracted as a single unit for each EVA, to ensure the best available information was captured. If needed, we would have contacted the authors of papers to request missing or additional data.

### Critical appraisal

As the purpose of this scoping review was to identify and explore the different methods used within reports contributing to EVAs rather than critique their overall applicability, appraisal of individual sources of evidence was not considered relevant to this research question and was not be undertaken. This is in line with accepted recommendations for conducting a scoping review (10).

### Data analysis and presentation

The unit of analysis in this review was individual Reports relating to EVAs. We described the data in tabular or graphical format with an accompanying narrative summary. We highlighted any commonalities in methods and briefly described areas of heterogeneity between reports. To do so, we reflected on guidance from the JBI to ensure the findings were accessible and interpretable. Insofar as possible, we reported the review in accordance with the PRISMA extension for scoping reviews (PRISMA-ScR) (12).

## Results

### Results of the search

**Figure 1.**
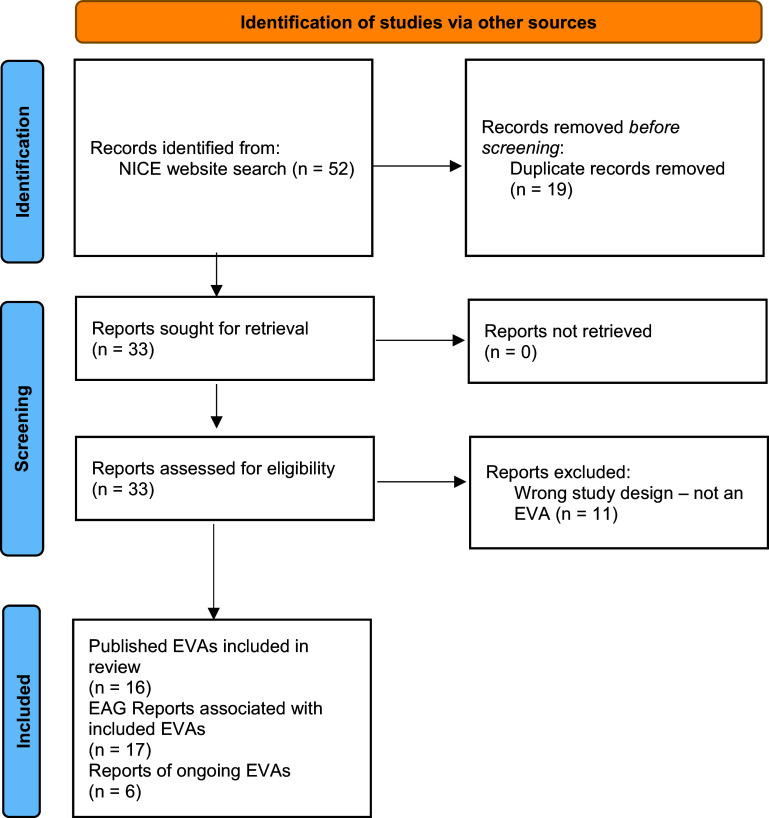
PRISMA flow diagram.

We retrieved fifty-two records from searching the NICE website, of which nineteen were duplicates. Following full-text assessment, eleven further reports were excluded. We included seventeen reports of sixteen EVAs and six reports of ongoing EVAs. One included EVA (HTE14) was initially conducted by one EAG, with a second, updated assessment and report conducted by another EAG. We included both Reports in this scoping review as separate entities on the same topic (13;14). The flow of literature is presented in the PRISMA diagram in Figure 1. Excluded reports with rationales are listed in Supplementary Material 5.

### General characteristics of reports for EVAs

Details of the included reports are presented in Table 1. In brief, twelve of the reports were of EVAs conducted as part of NICE’s Medical Technology Evaluation Program (13-19), whereas five were part of the Diagnostics Assessment Program (20-24). All but three of the reports were initially published prior to NICE’s interim statement on EVA methods (18;19;25). According to the final scopes issued by NICE, the number of interventions to be assessed within a single EVA ranged from one to fourteen (25-28), with the number of comparators ranging from one to twenty-one (13–16;18;20;21;23–26;29). Details on the purpose of interventions and comparators, as well as the primary population under consideration for each EVA report, are described in Supplementary Material 6. The number of proposed subgroups ranged from none to seventeen (15;20;21;23;29). The number of clinical-effectiveness outcomes in the NICE final scope ranged from ten to twenty-six (13-16;18;20;21;23-26;29), with the number of economic outcomes ranging from two to ten (13;14;22). One report did not specify any economic outcomes in the final scope but gave potential specifications for an economic model (15). Patient and public involvement was embedded into two reports (21;22). All but one report specified potential equity considerations (15).

**Table 1.** Characteristics of included EVAs

Reference	Undertaken as part of the following NICE workstream	Number of interventions	Number of comparators	Number of primary populations specified	Number of subgroups	Number of clinical outcomes	Number of economic outcomes	PPIE included in Report?	Equity considerations included?
HTE3 (26)	MTEP	5^a^	1	1	2	10	2	No	Yes
HTE4 (20)	DAP	1	1	1	3	10	6	No	Yes
HTE5 (15)	MTEP	1^b^	1	1	NR	13	0	No	No
HTE6 (21)	DAP	1	1	1	5	14	3	Yes	Yes
HTE7 (22)	DAP	8	2	2	17	11	5	Yes	Yes
HTE8 (16)	MTEP	7	21	1	2	17	2	No	Yes
HTE9 (17)	MTEP	11	2	1	3^c^	16	2	No	Yes
HTE10 (23)	DAP	1	1	2	1	16	5	No	Yes
HTE11 (27)	MTEP	11	4	1	NR	12	10	No	Yes
HTE12 (24)	DAP	14	1	2	4	20	5	No	Yes
HTE13 (29)	MTEP	1	1	2	2	19	2	No	Yes
HTE14a (13)	MTEP	8	1	1	None	20	2	No	Yes
HTE14b (14)	MTEP	12	1	1	None	10	2	No	Yes
HTE15 (28)	MTEP	4	7	1	3	15	4	No	Yes
HTE16 (18)	MTEP	8	1	1	2	26	3	No	Yes
HTE17 (25)	MTEP	3	1	1	3	19	4	No	Yes
HTE18 (19)	MTEP	6	4	1	4	10	5	No	Yes

*Note*: All characteristics are taken from the final scope of each EVA, as reported by NICE, except for whether PPIE was included in the EAG Report.

Abbreviations: DAP, Diagnostics Assessment Programme; EAG, Evidence Assessment Group; EVA, early value assessment; HTE, health technology evaluation; MTEP, Medical Technologies Evaluation Programme; NICE, National Institute for Health and Care Excellence; NR, not reported; PPIE, patient and public involvement and engagement.

a 4 plus standard care.

b One intervention, comprised of three modules.

c Not specified in the decision problem table but described as “varying levels of digital literacy or access, protected characteristics and comorbidities”.

### Methods for identifying clinical and economic evidence

**Figure 2.**
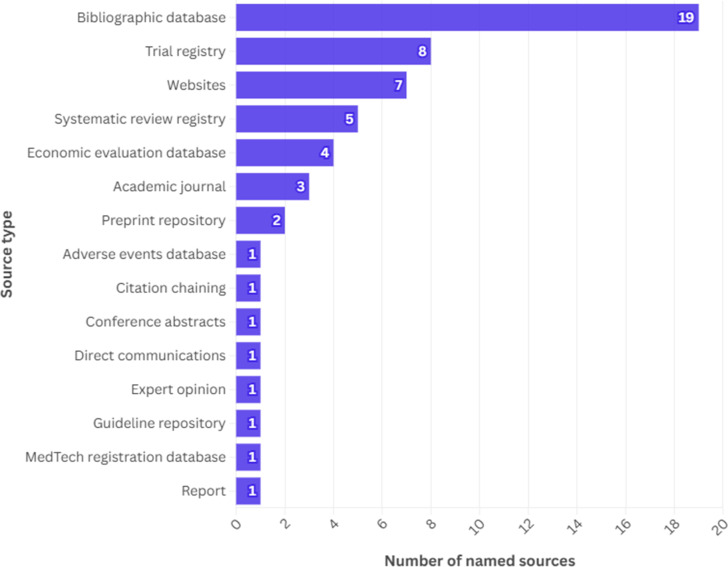
Categories of sources used to search for evidence and number of individual sources in each category.

As shown in Figure 2, a wide range of sources was used to search for evidence to inform EVAs. Nineteen different bibliographic databases, eight trial registries, and seven websites were used as data sources across the included reports. All seventeen included reports used MEDLINE and Embase as sources (13–29), whereas sixteen searched either ClinicalTrials.gov or the World Health Organization International Clinical Trials Registry Platform (WHO ICTRP) as sources of ongoing clinical studies. To identify economic evidence, five reports stated they used the Cost-Effectiveness Analysis (CEA) Registry (16;17;19;27;28), five used NHS Economic Evaluation Database (NHS EED; not updated since 2014) (17–19;28;29), and two used EconLit (18;29). A full list of specific sources used across the included reports is documented in Supplementary Material 7.

All but one of the included reports described using controlled vocabulary and free text terms within their search strategies; the remaining report did not describe how searches were constructed (24). No search limits were placed in nine reports (15;17;19;20;22;23;25;27;28), three restricted by language (16;24;26), two restricted both by date and to adult participants (13;14), one by both language and date (21), one by both study design and language (29), and one by study design, language and publication types (18). The following specific filters were used in reports: Centre for Reviews and Dissemination Health Economics filter (16); economic evaluation filter (13); NICE filters for MEDLINE and Embase (14); and NICE filter for health apps (18;19). Eleven reports did not report whether search filters were used (15;17;20–28). Nine of the reports conducted a single search for clinical- and cost-effectiveness evidence (16–19;23;25;26;28;29), with another three explicitly stating that they added economic evaluation filters to these searches (13;21;22).

### Screening and data extraction

Most included reports stated that one reviewer undertook screening (*N* = 11) (15–19;24–29), or data extraction (*N* = 12) (16–25;27;28). One report did not specify how many reviewers undertook screening (14), whereas five did not report how many undertook data extraction (13–15;26;29). Ten of the included reports did not specify whether the data extraction form was piloted (13–15;18;19;23;26–29), though the other seven stated that piloting took place (16;17;20-22;24;25).

Three reports did not provide the proportion of records checked by another reviewer at the screening stage (14;15;26), whereas six did not report on the proportion of extractions checked by another reviewer (13–15;26;29). In the remaining reports, the proportion of records checked by another reviewer varied, particularly when screening. However, six reports reported that all extractions were checked by a second reviewer (17;20–23;25). Eight reports stated doing complementary targeted searches to inform the economic model structure and parameters (13;15;20–22;24–26).

### Critical appraisal

Seven reports were either conducted or planned a critical appraisal. The following tools were employed to conduct a formal critical appraisal: the Cochrane “Risk of bias” tool (26); Cochrane “Risk of bias” 2 (22); Prediction model Risk Of Bias Assessment Tool (PROBAST) (20); Quality Assessment of Diagnostic Accuracy Studies-2 (QUADAS-2) (21–23); Risk Of Bias In Non-randomized Studies – of Interventions (ROBINS-I (21;22)); and the JBI tools (17;24). One report specified using the Consolidated Health Economic Evaluation Reporting Standards (CHEERS) 2022 checklist as a critical appraisal tool, despite its primary purpose being to assess completeness of reporting (26;30). Five reports stated that a single reviewer undertook a critical appraisal (20–24). A second reviewer checked 100 percent of all assessments in four of these reports; (20–23). the remaining report indicated that 20 percent of assessments were checked (24).

### Synthesis methods

Five reports indicated that they planned to conduct meta-analysis if feasible (17;21;22;24;25), nine planned narrative syntheses (16;17;19;20;23–25;28;29). and seven planned narrative summaries (13–15;18;21;22;27). One report did not explicitly state the method used to synthesize clinical effectiveness evidence, but presented a narrative summary (26). Fifteen reports conducted a narrative synthesis of economic evaluations (13;15–20;22–29); the remaining two did not describe how economic evaluations were synthesized (14;21).

### Economic model structure

Table 2 provides an overview of the approaches to economic modeling used across the included reports. In brief, the economic evaluations took the form of cost-utility analyses (CUA) in seven reports (13;14;17;18;21;26;28); cost-effectiveness analyses (CEA) in one (16); both a cost-consequence analysis and CEA in one (19); and cost-consequence in one (23). Six reports were unable to produce a full economic evaluation due to a lack of data (15;20;24;27). Finally, one report used a cost-consequence approach to model two interventions, but a CEA to model a third (25). A simplified coded model was feasible in all but six reports (14–17;20–22;24–26;28). Eleven reports presented a conceptual model (13;14;17;22;24;26). The most common approach was decision tree modelling in eight reports (16;21;25;28), followed by a Markov model in five (18;19;27). The type of decision model proposed was not presented explicitly in the three reports (21;22;29). Time horizons in the reports ranged from thirty days to lifetime (15;23;27); three did not report the time horizon (13;14;16–19;21;26;28;29). Methods used to assess uncertainty included: scenario analyses in ten EVAs (13;17–19;21;26;28;29); deterministic sensitivity analyses in eight (16;18;26;28;29); probabilistic sensitivity analyses in six (16–18;26;28;29); value of information in two (17;26); and an economically justifiable price in one (29). Seven reports did not report proposed methods for assessing uncertainty in model outputs (14;15;28;29). Only four reports explicitly validated model inputs with experts (20;22;23;27); four did not report whether inputs were validated (16–21;24;27–29). Ten reports did not specify the software proposed or used to build the coded model (13;14;25), three reported using TreeAge (13;14;25), two used Excel (15;23), and two used R (22;26).

**Table 2.** Overview of methods used to structure proposed economic models

Reference	Type of economic evaluation performed	Simplified coded model?	Conceptual model proposed?	Type of economic model	Description of how uncertainty was assessed	Were model predictions validated by experts?	Software used for model	Time horizon
HTE3 (26)	CUA	Yes	Yes	Decision tree	PSA, DSA, Scenario analysis, VOI (EVPI, EVPPI)	No	R	12 months
HTE4 (20)	Other: unable to run a full economic evaluation	No	Yes	Decision tree and state transition	NR	NR	NR	8 years
HTE5 (15)	Other: cost-comparison	No	Yes	Discrete event simulation	NR	Yes – inputs	Excel	NR
HTE6 (21)	CUA	Yes	Yes	Markov	DSA, Scenario analysis	No	NR	Lifetime
HTE7 (22)	Other: unable to run a full economic evaluation	No	Yes	Decision tree	NR	NR	R	Lifetime
HTE8 (16)	CEA	Yes	Yes	Markov	PSA, Scenario analysis	No	NR	2 years
HTE9 (17)	CUA	Yes	Yes	Decision tree	Scenario analysis, DSA, PSA, VOI (EVPI)	No	NR	15 months
HTE10 (23)	Cost-consequence analysis	No	No	NA	NR	NR	NR	NR
HTE11 (27)	Other: unable to run a full economic evaluation	No	No	Summative approach of costs and benefits	NA	NR	Excel	NR
HTE12 (24)	Other: cost-comparison	No	Yes	Decision tree	NR	No	NR	5 years
HTE13 (29)	Other: cost-comparison	Yes	No	Cost-comparison	PSA, DSA, Scenario analysis, EJP^a^	Yes – inputs	NR	30 days
HTE14a (13)	CUA	Yes	No	Decision tree	DSA, Scenario analysis	No	TreeAge	24 months
HTE14b (14)	CUA	Yes	Yes	Decision tree	Scenario analysis	Yes – inputs	TreeAge	24 months
HTE15 (28)	CUA	Yes	Yes	Markov	PSA, DSA, Scenario analysis	Yes – inputs	NR	5 years
HTE16 (18)	CUA	Yes	No	NR	PSA, DSA, Scenario analysis	No	NR	1 year
HTE17 (25)	AVATAR and SlowMo: cost-consequence; CareLoop: CEA	Yes	Yes	Markov	NR	No	TreeAge	AVATAR and SlowMo: 24 months, CareLoop: 3 years
HTE18 (19)	Cost consequence analysis and CEA	Yes	No	NR	DSA, Scenario analysis	No	NR	1 year

Abbreviations: CEA, cost-effectiveness analysis; CUA, cost-utility analysis; DSA, deterministic sensitivity analysis; EJP, economically justifiable price; PSA, probabilistic sensitivity analysis; NR, not reported; VOI, value of information; EVPI, expected value of perfect information; EVPPI, expected value of perfect parameter information.

a EJP meaning the maximum price that can be set for a health care intervention that results in an incremental cost-effectiveness ratio equal to the willingness-to-pay threshold (31).

### Methods used to determine outcomes and resource use for cost-effectiveness evidence

Table 3 provides an overview of the health effects and outcomes included in the analysis of economic evidence. As would be expected, the health effects and measurement tools described across the seventeen reports varied by topic area; these are reported in Supplementary Material 8. As shown in Table 3, methods used to derive costs and resource use were similarly heterogeneous. Only one report included carer costs (28), whereas none of the included reports included a severity modifier.

**Table 3.** Data sources for effectiveness, resource use, and cost used in the economic analysis

Reference	Data sources for effectiveness	Carers included?	Severity modifier?	Data sources for cost and resource use	Details of search strategies for cost and resource use	Cost categories considered
HTE3 (26)	Other: Results from the combined search of clinical and economic studies. Targeted search for HRQoL in depression	No	No	Company descriptions and assumptions	NR	Licencing costs Access device (tablet, computer, or smartphone) Data SIM card Telephone consultation with a therapist Telephone consultation with a clinical psychologist
HTE4 (20)	Rapid review and targeted searches	No	No	ORFAN 4 study	NR	Due to scarce evidence in health resource use impacts, cost categories relevant to the NHS and PSS could not be defined.
HTE5 (15)	Other: Additional hand searches, reference checks, and targeted searches. Additional inputs from clinical experts were used	No	No	Company estimates and expert opinion	NR	Cost of purchasing the intervention Cost per additional linear accelerator (linac) machine Cost per additional 1 TB of data storage Consultant oncologist time Peer review activity
HTE6 (21)	Other: Rapid review of cost-effectiveness and a targeted literature review	No	No	Estimates provided by the company and expert opinion	NR	Non-staff costs of implementation, staff costs (training and implementation), antibiotics, testing for hearing loss, hearing aids and cochlear implants
HTE7 (22)	Clinical effectiveness review and pragmatic searches	No	No	Published evidence from the main review and targeted search	NR	Cost of testing Staff time GP appointments Antibiotic courses Managing UTIs Managing UTI complications
HTE8 (16)	Other: Combined search of clinical and economic studies, particularly the economic model from NG222	No	No	Cost and resource use supplied by the companies from consultation responses	NR	Licence costs NHS support time Computing equipment and internet access Training and administration Oher costs
HTE9 (17)	Other: Update of the searches from a previous EVA (MT580). IAPT database used to model the comparator	No	No	Rapid review of clinical and economic studies	NR	Licence costs Therapist cost and time
HTE10 (23)	Rapid review	No	No	Company data	NR	Intervention costs consisted of software costs (including license and subscription costs) Hardware costs (one-off installation costs) Data storage costs Maintenance costs
HTE11 (27)	Other: Results from the scoping review	No	No	Unpublished pilot project reports	NR	Reading costs Interpretation costs Device set up costs
HTE12 (24)	Rapid review with complementary targeted searches. Post hoc search	No	No	Cost-effectiveness review; manufacturer cost data	NR	Costs are required for each AI software Costs for training staff to use software Resource use and costs associated with healthcare professional time to read and report CXR Costs for diagnostic testing and treatment.
HTE13 (29)	Other: Company estimates and expert elicitation	No	No	Company estimates and expert elicitation	NR	Virtual ward costs (licencing, tablets, set up, training, monitoring etc.) NHS staff NHS services
HTE14a (13)	Other: Combined search of clinical and economic studies, and a targeted search for HRQoL outcomes	No	No	Company estimates, clinical expert opinion, and previous EVAs	No additional cost search	Costs of technologies (licence cost) Digital inclusion (e.g. additional equipment) Weight management services
HTE14b (14)	Other: A previous EVA (HTE14a). An RCT (LIVA RCT) was used to inform standard care (32)	No	No	Company estimates and EVA HTE 10007	NR	Licence costs Participant additional costs
HTE15 (28)	Other: Results from the combined search of clinical and economic studies. Additional expert opinion on inputs	Carer costs	No	Company estimates, specialist committee member comments	NR	Licencing costs Therapist time and costs VR set capital costs
HTE16 (18)	Other: Clinical correspondence and company evidence submissions	No	No	Studies covering wider populations outside LBP. Resource use came from company estimates	NR	Device costs Primary care Physiotherapy referrals Secondary care Medications use
HTE17 (25)	Rapid review of clinical and cost-effectiveness evidence; complementary single-reviewer targeted search of economic evaluations on psychosis; additional unpublished data supplied by the companies	No	No	Company estimates	NR	System costs: set up, training costs, training time, additional hardware and infrastructure Costs per patient: Licensing, set up time, administrative time, HCP time per session, total HCP time, HCP cost, number of sessions, patient hardware, patient time
HTE18 (19)	Other: Results from the single clinical and economic search were used to inform effectiveness	No	No	Control arm costs come from one of the selected economic evaluations	NR	Licencing costs Health care professional costs Training Additional costs

Abbreviations: AI, artificial intelligence; CXR, chest x-ray; EVA, early value assessment; GP, general practitioner; HCP, healthcare professional; HTE, health technology evaluation; HRQoL, health-related quality of life; IAPT, *N*/A = not applicable; NHS, National Health Service; NR, not reported; RCT, randomized controlled trial; TB, terabyte; UTI, urinary tract infection; VR, virtual reality.

### Equity considerations

Of the fifteen reports where equity considerations were listed in the NICE final scope, only eight reported on methods for assessing equity (13;16–18;22;25;28;29). Of these eight, five reported that subgroup analyses had been planned to explore equity considerations, had data allowed (18;22;25;28;29). For the remaining three reports, two specifically listed digital inequalities as an outcome of interest (16;17), whereas one narratively reported on equity considerations (13).

## Discussion

### Summary of findings

We included seventeen reports across sixteen EVAs in this rapid scoping review. As expected, with a diverse range of clinical conditions being assessed, the methods used across the reports varied. There were often inconsistencies in the reporting of methods to assess clinical effectiveness across the seventeen reports, where the approaches used or planned were often not described. This was particularly with regard to the methods for study screening, data extraction, and syntheses. It should be noted that the lack of reporting of these methods does not mean that these steps did not take place or that the methods used within the reports were not systematic; it is that the approaches taken were not explicitly stated in the text.

Issues related to the lack of clarity in reporting search methods of clinical evidence impacted the searches for economic evidence, as reports generally relied on a single search of clinical effectiveness and economic evidence, often by applying economic filters to the clinical effectiveness search. Eight reports relied on an additional targeted or ad-hoc literature review to inform a simplified coded model to meet NICE’s and the DSU report’s recommendations (3;7). However, the reporting of the search strategies used for targeted literature reviews was not consistent across these reports.

NICE’s methods advise the use of expert opinion to validate models (3); the DSU report recommends that informal methods of expert elicitation can be used as a pragmatic tool to meet the resource and time limitations (7). Four reports explicitly stated validating their model inputs with expert opinion (14;15;28;29), the use of validation tools like the Assessment of the Validation Status of Health-Economic decision models (AdViSHE) was not reported (33).

Crucially, the data available to EAGs to conduct the reports was often limited. As the effectiveness evidence was often sparse and heterogeneous within the reports (e.g. HTE11 found a lack of data on patient outcomes at the time of review) (27).Specifically in decision problems with multiple comparators, this made it difficult to parametrize long-term treatment consequences. In six reports, the evidence gathered did not allow for a full economic evaluation. The lack of published data for treatment consequences on health care resource use meant that EAGs primarily relied on data supplied directly by the companies or obtained from clinical experts. Cost categories related to software implementation (training and expansion of the IT infrastructure in the NHS) overlapped in some reports. The carer’s perspective was included explicitly in the form of carer costs for HTE15 (28). Across the seventeen reports, the evidence available allowed for the construction of seven CUAs and three reports had at least an element of CEA in their analysis.

However, some reports went beyond the expectations of NICE’s interim methods guidance (3). Although NICE notes full critical appraisal is not required (Section title “*Critical appraisal*”), seven of the seventeen reports included in this review undertook or planned a full risk of bias assessment (3;17;20–24;26). Furthermore, although the interim methods statement notes that a quantitative meta-analysis is not expected (Section title “*Synthesis methods*”), five reports reported that they would have conducted meta-analyses if data allowed (3;17;21;22;24;25).

Executable models were presented either in Microsoft Excel, TreeAge or R. The software used or proposed to build the economic model was often not reported and generally not justified. Although this is not an essential step in the NICE methods, this choice can impact validation in coded models that are expected to be populated in the future when more data becomes available.

### Strengths and limitations of this review

This rapid scoping review has several strengths. Firstly, we prepublished the review protocol on the OSF (11). limiting the potential for bias. We took an inclusive approach to the eligibility of EVA reports, not limiting them by date or type of technology, to ensure we captured as many relevant reports as possible. Insofar as possible, we used the methods outlined by the JBI to conduct the review, including a completed PRISMA-ScR checklist for transparency (12;34). However, there were some limitations in the conduct of the review. Although two reviewers undertook screening for eligible EVA reports, data extraction was undertaken by a single reviewer and checked by a second. Although we took a pragmatic approach to review conduct, and we reflected on the latest guidance from the Cochrane Rapid Reviews Methods Group (35). it is possible that this may have increased the chance for inaccuracies when extracting data.

### Implications for practice and research

This scoping review found that methods used to inform the clinical effectiveness sections of EVA reports were inconsistently reported. In the short term, researchers may wish to consider the use of an appropriate checklist to guide this process. A PRISMA extension for rapid reviews, which would be most relevant to EVAs due to the eight-to-ten-week time frame for EAGs to complete their report, is currently under development (36). Though, as appropriate, researchers could adopt the PRISMA extension for Diagnostic Test Accuracy (PRISMA-DTA) or the full PRISMA statement to aid transparent reporting of methods (34;37). We also suggest the use and reporting of the Assessment of the Validation Status of Health-Economic decision models (AdViSHE) checklist as a tool to improve the transparency and standardization in the use of expert opinion to inform and validate decision model development, given project constraints (38).

NICE’s DSU acknowledges that the non-systematic identification of parameter values coupled with an over-simplification of the model structure risks misrepresenting the key drivers of value and may lead to erroneous conclusions about the potential cost-effectiveness of a technology (39). Although the approach to economic assessment in EVAs is meant to allow for flexibility, the lack of transparency and standardization in the reporting of searches for evidence means there is a risk that decisions are informed by a distorted or incomplete assembly of data.

More broadly, all the reports identified for this scoping review used what may be considered “traditional” methods of evidence synthesis and economic modeling that are often used when the evidence base is stronger, and more resources are available for analysis. However, EVAs are designed to promote the early adoption of medical technologies in the NHS at a time when the quality of data that can be used in an evidence synthesis or economic model may be very limited. As noted by NICE’s interim methods for EVAs, these assessments rely on emerging data and technologies in earlier phases of development, meaning that conducting informative systematic reviews and building economic models is often unachievable (3). The lack of published evidence often meant that EAGs strongly relied on evidence provided by manufacturing companies and, occasionally, clinical experts. Yet, it is uncertain whether the deadlines and requirements from the current EVA approach allow enough flexibility for an EAG to engage with key stakeholders, including patients and clinicians, companies, and NICE.

As such, EAGs need to reflect on which methods are most appropriate to answer individual research questions. Indeed, NICE’s interim statement on EVA methods notes that it is an overview of methods and processes, but is not designed to be prescriptive in detail, to allow flexibility (3). Tailored methods could help provide a greater understanding of the research landscape where data are limited. For example, evidence gap maps (EGMs) could provide a visual overview of the evidence base surrounding a topic, leading to easier identification of research gaps that could then feed into the evidence generation plan (40). A framework such as PROGRESS-Plus could be used to identify which factors potentially leading to health inequalities have been accounted for in the design and testing of the technologies (41;42).

Though the DSU currently advises pragmatic approaches given the nine-week deadline for EVAs, there may also be value in adopting methods such as discrete choice experiments, expert elicitation, and rapid qualitative methods to provide insights into patient and clinician preferences (7;43). Such preferences may have a direct impact on the adoption and implementation of health technologies, where standard measures of cost-effectiveness risk not fully capturing patient concerns (44;45).

As noted by a recent scoping review, (46) this work identified only two EVA reports that involved patients and the public (21;22). Wale et al. (47) posited that patients can help provide early scientific advice for health technology assessment (47), but, at least in the context of digital technologies, it has been noted that patients are often only involved in the latter stages of the innovation pipeline (e.g., usability testing) (48). EVAs could provide a unique opportunity to incorporate patient and public perspectives, as well as those of clinicians, into the design, implementation, and future evidence generation of emerging medical technologies.

We acknowledge the role of the EVA process in assessing the potential value of new technologies rather than reaching a final recommendation on adoption, which implies that methods need to remain flexible and transparent. However, to achieve this, there may need to be a shift towards greater collaboration between all relevant stakeholders to identify the most appropriate methodologies to assist the NICE committee in decision-making, as well as flexibility in how EAGs approach individual EVAs. As NICE states that the approaches outlined in their interim methods are iterative and can change to meet the needs of individual projects (3). This leaves the possibility of adopting innovative and collaborative methods in EVAs open.

## Conclusion

In this rapid scoping review, which included a total of seventeen published EAG reports across sixteen EVAs, we found inconsistencies in the reporting of the methods used. This was particularly the case for the reporting of methods used to assess clinical effectiveness, where some methodological details were either seldom reported or went beyond the requirements suggested by NICE in their interim guidance for EVAs (3). This had direct implications for the economic evidence as clinical and cost-effectiveness searches were generally conducted together, and informed the economic analysis and further conceptual model development. Future EAG reports for EVAs should aim for further transparency in reporting of methods, reflecting on NICE’s interim methods guide for conducting EVAs (3). The nature of EVAs also means there are opportunities to explore alternative and innovative methods of assessing the overall potential value of these medical technologies to the NHS, while further allowing for the inclusion of key stakeholders at this crucial stage of the process.

## Supporting information

Johnson et al. supplementary material Johnson et al. supplementary material

## Supplementary material

The supplementary material for this article can be found at http://doi.org/10.1017/S0266462325100433.
