# Supplementary material for: Methods used in early value assessments for nice: a scoping review
Source: Int J Technol Assess Health Care. 2025 Sep 8;41(1):e64. doi: 10.1017/S0266462325100433 (PMC12450547; doi:10.1017/S0266462325100433)
Supplement: Johnson et al. supplementary material [file S0266462325100433sup001.docx]

**Supplementary Material 1: Differences between protocol and review**

Here, we list the changes that we enacted during the review process that deviated from the prospectively published protocol.

*Search strategy*

- We amended the search terms to reflect that some EVAs are described as either a “health technology evaluation” or “health technology assessment”. As such, we added these terms to the searches to help ensure no EVAs were missed in the searching process.

*Data extraction*

- Following piloting of the original draft data extraction form, we made changes to the form to facilitate simpler data synthesis and comparison across included EVAs (e.g. adding further defined domains regarding methods for searches, screening, data extraction and critical appraisal). Full details of the final data extraction form can be found in Supplementary Material 4.
- We modified the process for data extraction. Pairs of independent reviewers extracted details from included EVAs; one pair extracted information for clinical effectiveness methods and the other pair extracted methods for cost-effectiveness analyses. This change was introduced in accordance with the skillsets of individual reviewers, to ensure greater accuracy of extraction.

**Supplementary Material 2: PRISMA-ScR checklist**

| **SECTION** | **ITEM** | **PRISMA-ScR CHECKLIST ITEM** | **REPORTED ON PAGE #** |
| --- | --- | --- | --- |
| **TITLE** | | | |
| Title | 1 | Identify the report as a scoping review. | Title page (1) |
| **ABSTRACT** | | | |
| Structured summary | 2 | Provide a structured summary that includes (as applicable): background, objectives, eligibility criteria, sources of evidence, charting methods, results, and conclusions that relate to the review questions and objectives. | 3 |
| **INTRODUCTION** | | | |
| Rationale | 3 | Describe the rationale for the review in the context of what is already known. Explain why the review questions/objectives lend themselves to a scoping review approach. | 4-5 |
| Objectives | 4 | Provide an explicit statement of the questions and objectives being addressed with reference to their key elements (e.g., population or participants, concepts, and context) or other relevant key elements used to conceptualize the review questions and/or objectives. | 5 |
| **METHODS** | | | |
| Protocol and registration | 5 | Indicate whether a review protocol exists; state if and where it can be accessed (e.g., a Web address); and if available, provide registration information, including the registration number. | 5 |
| Eligibility criteria | 6 | Specify characteristics of the sources of evidence used as eligibility criteria (e.g., years considered, language, and publication status), and provide a rationale. | 5-6 |
| Information sources* | 7 | Describe all information sources in the search (e.g., databases with dates of coverage and contact with authors to identify additional sources), as well as the date the most recent search was executed. | 5-6 |
| Search | 8 | Present the full electronic search strategy for at least 1 database, including any limits used, such that it could be repeated. | N/A |
| Selection of sources of evidence† | 9 | State the process for selecting sources of evidence (i.e., screening and eligibility) included in the scoping review. | 6 |
| Data charting process‡ | 10 | Describe the methods of charting data from the included sources of evidence (e.g., calibrated forms or forms that have been tested by the team before their use, and whether data charting was done independently or in duplicate) and any processes for obtaining and confirming data from investigators. | 6 |
| Data items | 11 | List and define all variables for which data were sought and any assumptions and simplifications made. | 6, Supplementary Material 4 |
| Critical appraisal of individual sources of evidence§ | 12 | If done, provide a rationale for conducting a critical appraisal of included sources of evidence; describe the methods used and how this information was used in any data synthesis (if appropriate). | 6 |
| Synthesis of results | 13 | Describe the methods of handling and summarizing the data that were charted. | 7 |
| **RESULTS** | | | |
| Selection of sources of evidence | 14 | Give numbers of sources of evidence screened, assessed for eligibility, and included in the review, with reasons for exclusions at each stage, ideally using a flow diagram. | 7-8 |
| Characteristics of sources of evidence | 15 | For each source of evidence, present characteristics for which data were charted and provide the citations. | 8-10 |
| Critical appraisal within sources of evidence | 16 | If done, present data on critical appraisal of included sources of evidence (see item 12). | N/A |
| Results of individual sources of evidence | 17 | For each included source of evidence, present the relevant data that were charted that relate to the review questions and objectives. | 11-22 |
| Synthesis of results | 18 | Summarize and/or present the charting results as they relate to the review questions and objectives. | 11-22 |
| **DISCUSSION** | | | |
| Summary of evidence | 19 | Summarize the main results (including an overview of concepts, themes, and types of evidence available), link to the review questions and objectives, and consider the relevance to key groups. | 22-23 |
| Limitations | 20 | Discuss the limitations of the scoping review process. | 23 |
| Conclusions | 21 | Provide a general interpretation of the results with respect to the review questions and objectives, as well as potential implications and/or next steps. | 23-25 |
| **FUNDING** | | | |
| Funding | 22 | Describe sources of funding for the included sources of evidence, as well as sources of funding for the scoping review. Describe the role of the funders of the scoping review. | 26 |

**Supplementary Material 3: Details of searches**

All searches were performed on the NICE website on 23 July 2024.

| **Search term** | **Number of records**  **retrieved** |
| --- | --- |
| Early value assessment (NICE guidance search - published) | 14 |
| Early value assessment (NICE guidance search - in development) | 4 |
| Early value assessment (NICE guidance search - in consultation) | 0 |
| Early value assessment (NICE guidance search - awaiting development) | 0 |
| Early value assessment (NICE guidance search - topic selection) | 0 |
| Health technology evaluation (NICE guidance search - published) | 18 |
| Health technology evaluation (NICE guidance search - in consultation) | 0 |
| Health technology evaluation (NICE guidance search - in development) | 13 |
| Health technology evaluation (NICE guidance search - awaiting development) | 2 |
| Health technology evaluation (NICE guidance search - topic selection) | 0 |
| Health technology assessment (NICE guidance search - published) | 1 |
| Health technology assessment (NICE guidance search - in consultation) | 0 |
| Health technology assessment (NICE guidance search - in development) | 0 |
| Health technology assessment (NICE guidance search - awaiting development) | 0 |
| Health technology assessment (NICE guidance search - topic selection) | 0 |
| **Total** | **52** |
| **Total following deduplication** | **33** |

**Supplementary Material 4: Items for data extraction**

| **Domain** | **Item** |
| --- | --- |
| Study characteristics | - Title - NICE ID - URL - Month and year published - Workstream (medical technologies, digital technologies, diagnostics, other) |
| Intervention | - Number of interventions assessed - Purpose of interventions assessed |
| Comparators | - Number of comparators assessed - Types of comparators assessed |
| Population | - Description of primary population - Description of subgroups considered |
| Outcomes | - Number of outcomes listed in final scope |
| Search strategies | - Data sources searched (e.g. MEDLINE, Embase) - Methods used to construct search strategies (e.g. MeSH terms, free text terms) - Details of any limitations (e.g. date, language) - Details of any filters applied (if applicable) - Brief details of methods used to search for ongoing studies - Any other relevant information regarding the searches |
| Study selection | - Software used for screening (e.g. Rayyan) - Number of reviewers assessing titles/abstracts and full texts for inclusion - Percentage of records checked by a second reviewer (if applicable) - How screening conflicts were resolved - Any other relevant information regarding study selection |
| Data extraction | - Software used for data extraction (e.g. Excel) - Did piloting take place? (yes/no) - Number of reviewers extracting data - Percentage of records checked by a second reviewer (if applicable) - How extraction conflicts were resolved |
| Critical appraisal | - Was critical appraisal undertaken? (yes/no) - If yes, what tool was used for critical appraisal? - Number of reviewers undertaking critical appraisal - Percentage of records checked by a second reviewer (if applicable) - How conflicts were resolved - Any other details regarding critical appraisal |
| Synthesis methods | - Was formal synthesis planned or undertaken? (yes, yes – planned, no) - Brief details of synthesis methods used or planned - Any other details regarding synthesis |
| Cost-effectiveness evidence | - Type of economic evaluation: fully incremental cost-utility analysis or cost-comparison or other - Time horizon - Brief details of strategies of relevant economic evaluations - Brief details of methods used for critical appraisal of economic evaluations - Methods used for synthesis of economic evaluations (narrative synthesis/ other)   - If ‘other’, brief details of synthesis method - Data sources of effectiveness evidence: systematic review/other   - If ‘other’, brief details of sources used - Search result details - Search strategy details - Health effects/outcomes included - Method of measuring benefit/quality of life (If QALYs, EQ-5D or other) - Methods for measuring quality of life - Carers included (yes/no) - Severity modifier (yes/no) - Data sources of costs and resource use (systematic review, other)   - If ‘other’, brief details of sources used - Cost categories considered - Search strategies of cost and resource use - Economic model used (yes/no)   - If yes: Type of economic model (decision tree, Markov, partitioned state model, other, N/A)   - If diagnostic: intermediate outcomes used   - How was uncertainty assessed (Probabilistic Sensitivity Aanalysis, scenario analysis, sensitivity analysis, value of information)   - Were model predictions validated by experts (yes/no)   - Model software (Excel, TreeAge, R, other) - Conceptual model? (yes/no) |
| Patient and public involvement and engagement (PPIE) | - Was PPIE undertaken? (yes/no)   - If yes, brief description of PPIE undertaken |
| Equity considerations | - Are equity considerations part of the NICE final scope? (yes/no) - If yes, are methods by the EAG to consider equity described? (yes/no) - If yes, brief details of methods used to consider equity |
| Abbreviations: ID = identifier; NICE = National Institute for Health and Care Excellence; PPIE = patient and public involvement and engagement; URL = uniform resource locator | |

**Supplementary Material 5: List of excluded reports**

| **Title** | **Reason for exclude** |
| --- | --- |
| Bed frames for patient care and recovery: late stage assessment | Wrong study design: not an EVA |
| Blinatumomab with chemotherapy for consolidation treatment of Philadelphia-chromosome-negative CD19-positive B-precursor acute lymphoblastic leukaemia with no measurable residual disease ID6405 | Wrong study design: not an EVA |
| Cefiderocol for treating severe drug-resistant gram-negative bacterial infections | Wrong study design: not an EVA |
| Ceftazidime with avibactam for treating severe drug-resistant gram-negative bacterial infections | Wrong study design: not an EVA |
| Compression products for wound care: late stage assessment | Wrong study design: not an EVA |
| Drug-eluting coronary stents for treating coronary artery disease: late-stage assessment | Wrong study design: not an EVA |
| Intermittent catheters for urinary management in adults : Late Stage Assessment | Wrong study design: not an EVA |
| One-piece closed bags for adults with a colostomy: Late Stage Assessment | Wrong study design: not an EVA |
| Slide sheets for repositioning or moving a person on or from a bed: Late Stage Assessment | Wrong study design: not an EVA |
| Topical Antimicrobial dressings for wound care: Late stage assessment | Wrong study design: not an EVA |
| Transcatheter heart valves for transcatheter aortic valve implantation in people with aortic stenosis: Late stage assessment | Wrong study design: not an EVA |

**Supplementary Material 6: Description of eligible interventions, comparators and primary populations in included EVAs**

| **Reference** | **Purpose of interventions** | **Purpose of comparators** | **Description of primary population(s)** |
| --- | --- | --- | --- |
| HTE3 | Guided digital self-help CBT technologies, supported by healthcare professionals | Standard care May include education, advice, support and signposting | Children (5 to 11) and young people (12 to 18) with mild to moderate symptoms of anxiety or low mood that are significantly interfering with their ability to function in their daily lives |
| HTE4 | To estimate individual patient risk of 8-year cardiac death with a prognostic model, which includes the perivascular FAI score, atherosclerotic plaque burden and clinical risk factors (used as an add-on to CTCA scans) | Current standard of care (CTCA without the addition of CaRi-Heart, alongside clinical risk assessment and patient-appropriate risk factor management | People with stable, recent onset chest pain, of suspected cardiac origin, who are undergoing CTCA. |
| HTE5 | To compare radiotherapy plans and enable collection of imaging and dosimetric data through a cloud-based data repository, communication and analytics software | Standard care | People having planned radiotherapy with 3D dose distribution |
| HTE6 | To detect the MT-RNR1 m.1555A>G variant | Not to test to determine the baby's MT-RNR1 m.1555 variant status before treatment with aminoglycosides | Any babies being considered for treatment with aminoglycosides |
| HTE7 | To detect and diagnose potential UTIs | 1. Dipstick testing, then laboratory-based testing (if necessary)  2. Laboratory testing alone | 1. People with suspected UTI who would have an initial dipstick test in current practice  2. People with suspected UTI who would not have an initial dipstick test in current practice |
| HTE8 | Digitally enabled therapies for facilitating guided self-help in people with depression, designed to be used with support by healthcare professionals | Standard care according to NICE's clinical guideline on depression in adults: treatment and management  Split into 11 options for less severe depression and 10 for more severe depression | Adults with depression who have been referred to IAPT services |
| HTE9 | Digitally enabled therapies delivered with the support of a practitioner or therapist | Standard care within the IAPT care pathway at low intensity (step 2) or high intensity (step 3) | Adults (18 years and older) with anxiety disorders (body dysmorphic disorder, GAD, health anxiety, OCD, Panic disorder with/without agoraphobia, PTSD, social anxiety disorder, specific phobias) |
| HTE10 | To measure QT interval in people having antipsychotic medication | Traditional 12-lead ECG performed in primary or secondary care | 1. Adults indicated for an ECG prior to starting antipsychotic medication 2. Adults taking antipsychotic medication who require ECG to monitor QT interval |
| HTE11 | To aid contouring for radiotherapy treatment planning | Contouring methods in standard care; no contours or no contouring | People having radiotherapy treatment planning for external beam radiotherapy |
| HTE12 | To analyse CXR for suspected lung cancer | Review from an appropriate radiology specialist without assistance from AI software | 1. People referred from primary care who are having an CXR because they have symptoms suggestive of lung cancer  2. People referred from primary care having CXR for reasons unrelated to lung cancer |
| HTE13 | To treat adults with acute respiratory infections as an alternative to inpatient hospital care | Inpatient hospital care or care in the community or a patient's usual place of residence without the use of a virtual ward platform | 1. Adults (aged 16 or over) referred for hospital admission with acute respiratory infection  2. Adults (aged 16 or over) admitted to hospital with acute respiratory infection who are stable or improving but require active monitoring |
| HTE14a | Digitally enabled weight management programmes providing specialist weight management services such as tier 3 or tier 4 services to support treatment with weight management medication | Standard care which could include specialist weight management services (including tier 3 and 4; face to face, remote or hybrid) alongside treatment with weight management medication; or no treatment or waiting list | Adults with obesity referred for treatment with weight management medication in line with NICE's guidance  Subgroups: None |
| HTE14b | Digitally-enabled weight management programmes to provide specialist weight management services (e.g. tier 3 or 4) for adults with obesity | Standard care, which could include specialist weight management services, no treatment or waiting list | Adults eligible for treatment in specialist weight management services (tiers 3 or 4), including people eligible for weight management medication |
| HTE15 | To treat agoraphobia or agoraphobic avoidance | Standard care, which may include: guided self-help; CBT; exposure therapy; applied relaxation; antidepressants licensed for treating panic disorder; oral antipsychotic medication; simple contact and monitoring with services | People aged 16 and over with agoraphobia or agoraphobic avoidance |
| HTE16 | Self-management and/or psychological support for the treatment of non-specific LBP | Standard care for managing LBP | People aged 16 years and over with non-specific LBP that are eligible for digital technology management |
| HTE17 | To provide specialist support for managing symptoms of psychosis or to prevent relapse in people with psychosis who are receiving care from healthcare professionals | Current standard of care For managing symptoms, this includes CBT, psychological support whilst waiting for CBT, and no access to psychological support No further details provided for managing people at risk of relapse | People aged 14 and over living with primary psychosis |
| HTE18 | Digitally supported pulmonary rehabilitation technologies | Standard care face to face pulmonary rehabilitation either in clinical or home-based setting; No treatment or waiting list   If data are available: hybrid face to face and remote live pulmonary rehabiliation;  non-digital non-face to face options for components of pulmonary rehabiliation e.g printed exercise sheets | Adults with confirmed diagnosis of COPD who: have had a recent hospitalisation because of acute exacerbation or whose functional baseline has greatly changed and is not following the expected recovery path; or have an MRC dyspnoea score of 2 or above; or have decreased exercise capacity as measured by a validated outcome measure such as 6min walk test |
| Note: All characteristics are taken from the final scope of each EVA, as reported by NICE  Abbreviations: CBT = cognitive behavioural therapy; COPD = chronic obstructive pulmonary disorder; CTCA = computed tomography coronary angiography; CXR = chest x-ray; ECH = electrocardiogram; FAI = ; GAD = generalised anxiety disorder; HTE = health technology evaluation; IAPT = ; LBP = lower back pain; MRC = Medical Research Council; N/A = not applicable; NICE = National Institute for Health and Care Excellence; OCD = obsessive compulsive disorder; PTSD = post-traumatic stress disorder; UTI = urinary tract infection; | | | |

**Supplementary Material 7: Specific sources searched within the included EVAs**

| **Source type** | **Name of source** | **Number of times used in included EVAs** |
| --- | --- | --- |
| Academic journal | European Heart Journal - Digital Health | 1 |
|  | International Journal of Medical Physics Research and Practice by the American Association of Physicists in Medicines | 1 |
|  | Journal of Applied Clinical Medical Physics from American Association of Physicists in Medicines (AAPM) | 1 |
| Adverse events database | Manufacturer and User Facility Device Experience (MAUDE) | 6 |
| Bibliographic database | Embase | 17 |
|  | MEDLINE | 17 |
|  | Cochrane Central Register of Controlled Trials (CENTRAL) | 12 |
|  | International Network for Agencies for Health Technology Assessment (INAHTA) database | 11 |
|  | Cumulative Index to Nursing and Allied Health Literature (CINAHL) | 5 |
|  | Epistemonikos | 5 |
|  | PsycINFO | 5 |
|  | ScanMedicine | 5 |
|  | PubMed | 4 |
|  | Database of Abstracts of Reviews of Effects (DARE) | 3 |
|  | National Institute for Health and Care Research (NIHR) database | 3 |
|  | Conference Proceedings Citation Index – Science (CPCI-S) | 2 |
|  | Centre for Reviews and Dissemination Health Technology Assessment (CRD HTA) database | 2 |
|  | Directory of Open Access Journals (DOAJ) | 2 |
|  | Latin American and Caribbean Health Sciences Literature (LILACS) | 2 |
|  | Association for Computing Machinery (ACM) Digital library | 1 |
|  | Centre for Reviews and Dissemination (not specified) | 1 |
|  | PubMed not MEDLINE | 1 |
| Citation chaining | Company submission references | 2 |
| Conference abstracts | Northern Light Life Sciences Conference | 2 |
| Direct communications | Communication with companies | 2 |
| Economic evaluations database | Sheffield Centre for Health and Related Research Health Utilities Database (ScHaRRHUD) | 5 |
|  | Cost Effectiveness Analysis (CEA) Registry | 5 |
|  | National Health Service Economic Evaluations Database (NHS EED) | 4 |
|  | EconLit | 2 |
| Expert opinion | Clinical experts | 1 |
| Guideline repository | Scottish Intercollegiate Guidelines Network (SIGN) | 5 |
| Medical technology registration database | Food and Drug Administration (FDA) services database | 1 |
| Preprint repository | MedRixiv | 5 |
|  | Engrxiv | 1 |
| Report | HTA (not specified) | 1 |
| Systematic review registry | Cochrane Database of Systematic Reviews (CDSR) | 12 |
|  | International prospective register of systematic reviews (PROSPERO) | 3 |
|  | Kleijnen Systematic Reviews (KSR) Evidence | 2 |
|  | International Platform of Registered Systematic Review and Meta-analysis Protocols (INPLASY) | 2 |
|  | Database of Abstracts of Reviews of Effects (DARE) | 1 |
| Trial registry | ClinicalTrials.gov | 16 |
|  | World Health Organization International Clinical Trials Registry Platform (WHO ICTRP) | 16 |
|  | European Union Drug Regulating Authorities Clinical Trials Database (EudraCT) | 2 |
|  | Australian New Zealand Clinical Trials Registry (ANZCTR) | 1 |
|  | Chinese Clinical Trial Registry | 1 |
|  | German Clinical Trials Register (DRKS) | 1 |
|  | European Union Clinical Trials Register (EUCTR) | 1 |
|  | ISRCTN (originally International Standard Randomised Controlled Trial Number) | 1 |
| Website | Company websites | 7 |
|  | Medicines and Healthcare Products Regulatory Agency (MHRA) | 5 |
|  | National Institute for Health and Care Excellence (NICE) | 5 |
|  | Google Scholar | 3 |
|  | National Institute for Health and Care Research Health Technology Assessment (NIHR HTA) programme | 2 |
|  | Future NHS virtual wards network | 1 |
|  | Google Advanced Search | 1 |

**Supplementary Material 8: Health outcomes and immediate outcomes for diagnostics in included EVAs**

| **Reference** | **Health effects/ outcomes included** | **If diagnostic, description of immediate outcomes used** | **Method of measuring health effects** | **Methods for measuring QoL** |
| --- | --- | --- | --- | --- |
| HTE3 | Differences in RCADS-P t-scores  HRQoL | N/A | RCADS-P | Targeted literature search and assumptions |
| HTE4 | Specificity and sensitivity of using CaRi-Heart and CTCA scans in combination.  Health state utilities. | Lack of evidence about the clinical effects of the intervention or of any changes in treatment/management from it | Proposed only: any published evidence using EQ-5D | N/A |
| HTE5 | Impact of changes and compliance to radiotherapy plans on overall survival and disease control | N/A | Patient outcomes in overall survival and disease control, changes in current radiotherapy plans | N/A |
| HTE6 | HRQoL  Cases of AIHL avoided | Diagnostic accuracy of Genedrive for detecting MT-RNR1  AIHL cases avoided | Diagnostic accuracy | Targeted literature search: estimates from a previous NICE TA |
| HTE7 | Complicated or uncomplicated UTIs  Antibiotics courses  Adverse events  HRQoL | Diagnostic accuracy for detecting a UTI | Diagnostic accuracy for detecting UTIs | Literature estimates of HRQoL |
| HTE8 | Depression severity using PHQ-9 or BDI scale thresholds.  Response to treatment  Discontinuation using log odds ratios. | N/A | PHQ-9, BDI-I, and BDI-II scores mapped | EQ-5D estimates from NG222 for utility values |
| HTE9 | GAD-7  Recovery rates  HRQoL | N/A | GAD threshold define recovery/non-recovery, incremental QALYs | Assumptions |
| HTE10 | NR | Diagnostic accuracy, where the target condition is QTc | Diagnostic accuracy | Scoping review results |
| HTE11 | Time taken to delineate organs at risk and review and edit images associated with AI auto-contouring (vs manual or atlas) was considered an outcome of economic interest since this could be quantified | Time taken to delineate organs at risk and review and edit images associated with AI auto-contouring (vs manual or atlas) was considered an outcome of economic interest | Seven studies identified measured time from the use of AI auto-contours, with heterogeneous study designs, heterogeneous time outcomes, and lack of consequence data for some technologies | N/A |
| HTE12 | Long term morbidity and mortality.  Intermediate outcomes: accuracy to detect lung cancer, turnaround time, technical failure rate, impact on decision-making, CT scan referral numbers, follow-up CXR referral numbers, number of cancers missed/detected, stage of cancer at detection, time to CT scan, time to CXR report, time to diagnosis, ease of use by clinicians | Accuracy to detect lung cancer (sensitivity and specificity) and turnaround time (from of image review to radiology report).  Secondary outcomes:  Technical failure rate  Impact on decision-making  Referrals to CT scans  Referrals to follow up CXR  Number of cancers missed/detected  Cancer stage at detection  Time to CXR report  Time to CT scan  Time to diagnosis  Ease of use | NA | N/A |
| HTE13 | None included, cost-minimisation | N/A | NA | N/A |
| HTE14a | Losing less than 5% body weight  Losing 5% or more body weight  Treatment discontinuation  HRQoL | N/A | Proportion achieving less than 5% body weight loss, proportion achieving 5% or more body weight loss, impact of body weight loss on HRQoL | EQ-5D-3L |
| HTE14b | Change in weight  Adherence and attrition  Adverse events  Health care resource use  Accessibility  Change in BMI  QoL  Patient experience  Psychological outcomes | N/A | Incremental QALYs | Assumptions |
| HTE15 | Probability of response  HRQoL  Relapse rates | N/A | Probability of response to treatment  Risk of relapse | Literature estimates of HRQoL |
| HTE16 | HRQoL  Pain scores  Work days missed due to LBP | N/A | Incremental utilities. Absenteeism (work days missed due to low back pain) | EQ-5D-3L EQVAS Mapping from pain-measurement instruments |
| HTE17 | Symptom management: Change in targeted psychotic symptoms, health related quality of life, patient experience, adherence, and adverse events.  Relapse prevention: rates of relapse, time to relapse, relapse severity, adherence, patient experience, adverse events, HRQoL | N/A | CareLoop and AVATAR: Utilities, EQ-5D-5L  SlowMo: unknown | EQ-5D-5L  PSYRATS |
| HTE18 | Exercise capacity  Respiratory function  HRQoL  Adverse events  Hospitalisations or ED visits  Cost per change in exercise capacity | N/A | Exercise capacity from 6MWD and ISWD | EQ-5D-5  EQ-5D-VAS  SGRQ  CRQ |
| Abbreviations: 6MWD =6-minute walk distance; AI = artificial intelligence; AIHL = aminoglycoside-induced hearing loss; BDI = Beck Depression Inventory; BDI-I = Beck Depression Inventory-I; BDI-II = Beck Depression Inventory-II; BMI = body mass index; CRQ = Chronic Respiratory Questionnaire; CT = computerised tomography; CTCA = computerised tomography coronary angiogram; CXR = chest x-ray; ED = emergency department; EQ-5D-VAS = EQ-5D visual analogue scale; EVA = early value assessment; GAD-7 = Generalised Anxiety Disorder Questionnaire-7; HRQoL = health-related quality of life; HTE = health technology evaluation; ISWD = Incremental Shuttle Walk Test; LBP = lower back pain; N/A = not applicable; PHQ-9 = Patient Health Questionnaire-9; PSYRATS = Psychotic Symptom Rating Scales; QALY = quality-adjusted life year; QoL = quality of life; RCADS-P = Revised Children’s Anxiety and Depression Scale-Parent; SGRQ = St George’s Respiratory Questionnaire; TA = technology appraisal; UTI = urinary tract infection | | | | |
